# Genetic Diversity Analysis of Mitochondrial *Cytb* Gene, Phylogeny and Phylogeography of Protected Griffon Vulture (*Gyps fulvus*) from Serbia

**DOI:** 10.3390/life12020164

**Published:** 2022-01-22

**Authors:** Slobodan Davidović, Saša Marinković, Mila Kukobat, Milica Mihajlović, Vanja Tanasić, Irena Hribšek, Marija Tanasković, Marina Stamenković-Radak

**Affiliations:** 1 Department of Genetics of Populations and Ecogenotoxicology, Institute for Biological Research “Siniša Stanković”—National Institute of the Republic of Serbia, University of Belgrade, Bulevar Despota Stefana 142, 11060 Belgrade, Serbia; marija.tanaskovic@ibiss.bg.ac.rs (M.T.); marina@bio.bg.ac.rs (M.S.-R.); 2 Department of Ecology, Institute for Biological Research “Siniša Stanković”—National Institute of Republic of Serbia, University of Belgrade, Bulevar Despota Stefana 142, 11060 Belgrade, Serbia; grifon@ibiss.bg.ac.rs; 3 Faculty of Biology, University of Belgrade, Studentski trg 16, 11000 Belgrade, Serbia; mmkukobat@gmail.com; 4 Center for Forensic and Applied Molecular Genetics, Faculty of Biology, University of Belgrade, Studentski trg 16, 11000 Belgrade, Serbia; milica.mihajlovic@bio.bg.ac.rs (M.M.); vanja.tanasic@bio.bg.ac.rs (V.T.); 5 Birds of Prey Protection Foundation, Bulevar Despota Stefana 142, 11060 Belgrade, Serbia; irena.hribsek@nhmbeo.rs

**Keywords:** Griffon vulture, *Cytb*, population genetics, conservation, Balkan Peninsula, endangered species

## Abstract

Once a widespread and common species across the region of southeast Europe, the Griffon vulture is now confined to small and isolated populations across the Balkan Peninsula. The population from Serbia with 290 couples represents its biggest and most viable population that can serve as an important reservoir of genetic diversity from which the birds can be used for the region’s reintroduction or recolonization programs. To estimate the level of genetic diversity, the mitochondrial *Cytb* gene from 58 unrelated birds sampled during the marking in the nests was sequenced and compared to the homologous Griffon vulture sequences available in publicly accessible online databases. Phylogeographic analysis based on *Cytb* sequences showed that the most frequent haplotype is found in all Griffon vulture populations and that each population possesses private haplotypes. Our data suggest that the Griffon vulture population from Serbia should be used as a source population for restocking and reintroduction programs in the region. The observed genetic differentiation between the populations from the Iberian and Balkan Peninsulas suggest that the introduction of foreign birds from remote populations should be avoided and that birds from indigenous or neighboring populations, if available, should be used instead.

## 1. Introduction

The Eurasian Griffon vulture (*Gyps fulvus fulvus* Hablizl, 1783) belongs to the subfamily *Aegypiinae* or *Gypiinae*, a group of Old World vultures that is listed as critically endangered on the International Union for Conservation of Nature’s Red List of Threatened Species (IUCN Red list) [1] and is an object of several international conservation conventions and directives (reviewed in [2,3]). Of 16 Old World vulture species, 11 are classified as “Globally Threatened”, with 8 “Critically Endangered” and 3 “Endangered” species. The causes of the worldwide decline of vulture populations are mainly due to the negative anthropogenic influence, which includes habitat destruction and degradation, human disturbance of nesting sites and changes in livestock management, as well as the disappearance of nomadic grazing on natural pastures, electrocution, windmills, poaching, use of non-steroidal anti-inflammatory drugs and poisoning [4]. Many conservative strategies around the globe are successfully implemented, especially in Europe [5,6,7,8,9], but the coordination and implementation of the global action plan still remains a necessity [3,4].

Vultures play a key role in maintaining the harmonious processes and functionality of ecosystems. As obligate scavengers, their main role is the decomposition of organic matter in nature, via efficient carcass removal that reduces the rates of transmission of infectious diseases. The Griffon vulture itself is an economically and ecologically important species that provides several ecosystem services in the form of animal carcass removal, which is beneficial from both the sanitary and economic perspective [10,11,12]. It is estimated that the Spanish vulture population, where the Griffon vulture is the dominant species, on average removes between 134 and 201 tons of bones and between 5551 and 8326 tons of meat each year, which allows for a minimum economic savings of approximately USD1.19 million to USD1.94 million [10]. Apart from this crucial ecosystem service that the vultures provide, the Griffon vulture is recognized as an important species that can improve the income of the region through ecotourism since the nature reserves that host this magnificent bird attract a lot of bird watchers [13,14,15]. The recognized role of the Griffon vulture in nature, as well as its economic value, makes this species important for protection and conservation efforts. Due to the all above mentioned reasons, as well as species recent predicaments, it is necessary to properly evaluate the existing genetic diversity of different Griffon vulture populations in order to design proper conservation strategies [16].

The Eurasian Griffon vulture has a large area of distribution, ranging from Kazakhstan and Nepal to Southern Europe and the Maghreb countries [17] and is the most widespread species of *Gyps* genus. Spain holds the largest breeding Griffon vulture population in the world. Estimates are that around 75% (about 26,000 couples) of the world’s total Griffon vulture population and more than 95% of European populations are situated in this country [18,19,20]. In the Balkan Peninsula, there are an estimated number of 550 couples, with the exclusion of the population from Crete [21]. The latest census estimate of the Griffon vulture population from Serbia for the year 2021 is 290 couples, which accounts for more than half of the couples estimated for the entire Balkan Peninsula [22]. The Griffon vulture population of the Balkan Peninsula consists of several subpopulations (addressed as populations further in the text) including those from Serbia, Croatia, Bulgaria, Greece and North Macedonia (reviewed in [3]), while in Europe, several isolated island populations in Sardinia, Cyprus and Crete exist (reviewed in [3]). According to the IUCN criterion, this species is currently classified as “Least Concern” due to its large population in Spain. However, bearing in mind that this species is now considered extinct from most parts of North Africa and Apennines [23] and several parts of its historical range in southeast Europe (Albania and Romania), protection and conservation of its remaining natural breeding colonies remain a necessity. Furthermore, the species is classified as a sensitive and dependent species and as such it is protected by various laws, directives and conventions. Genetic diversity of the Griffon vulture populations across its distribution range was evaluated in several studies and it is described on both biparentally inherited autosomal loci and its microsatellites [2,5,24] and uniparentally inherited mitochondrial DNA (mtDNA) [16,25,26,27,28,29]. The study [5], based on the variability of microsatellites, demonstrated a need for proper evaluation of genetic variability in native populations used for restocking and post-released populations in order to maintain the genetic diversity of reintroduced populations and preserve their stability. Further studies based on microsatellite variability demonstrated the existence of two distinct genetic clusters [2]. The first one is characteristic for populations from the Balkan Peninsula and another one for the population of the Iberian Peninsula, while the population from the Middle East showed the admixture of both genetic clusters. These new data are of great importance for future reintroduction projects [2], foremost because the study included the populations from the Balkan Peninsula for the first time. An earlier study demonstrated no genetic differentiation among the Griffon vulture populations from Spain, Israel and Cyprus, but it did not include specimens from the Balkan Peninsula populations [24]. The same study showed the lack of genetic diversity in the population of Indian vulture (*Gyps indicus*), which called for the proper genetic evaluation of birds that will be used for future restocking programs [24], further stressing the need for an accurate genetic characterization of the populations of conservation interest.

Considering mtDNA, the majority of the available data for different Griffon vulture populations are based on the analysis of *Cytb* [16,25,26,29] and D-loop [27,28]. In addition to the available data the first complete mitogenome was reported in [28], which allowed for a more accurate reconstruction of the evolutionary history of Griffon vulture, especially with regard to molecular dating. *Cytb* data were mostly used to determine the phylogeny of different vulture species, including the members of the *Gyps* genus [16,26,29], while only one study used *Cytb* for both phylogenetic and phylogeographic analysis [25]. A highly variable and selectively neutral D-loop region of mtDNA was used recently in studies whose goal was to determine the population genetic history of the isolated island Griffon vulture populations from Sardinia, Crete and Cyprus [27,28]. While substantially adding to the body of knowledge about the phylogeny of different vulture species, most of these studies, with the exception of [27,28], lack the population part of studying genetic diversity in Griffon vulture, which is of crucial importance for proper design of conservation, restocking and reintroduction strategies as well as establishing the appropriate scale and units for conservation management.

Properly designed conservation strategies recognize the need to analyze information from both uniparentally and biparentally inherited genetic markers [30,31,32]. Unlike genetic markers from autosomal loci, uniparental markers such as mtDNA and sex chromosomes (Y in mammals and W in birds) have a four times smaller effective population size [33,34], which makes them more vulnerable to genetic drift and rapid demographic change [35]. These characteristics of mtDNA are important when the conservation efforts are made for the populations that became highly divergent due to the long-term isolation [36] or have experienced a serious bottleneck event that can create enough genetic differences for evolutionary significant units (ESU) to be recognized [37]. This is the case with the Balkans’ Griffon vulture population from Serbia [2]. Analysis of mtDNA variability can also provide an insight into the reproductive behavior of the organisms, i.e., the identification of strong natal philopatric behavior [32,38], which is essential information for creating a proper conservation strategy. The mitochondrial *Cytb* gene is widely used in studies considering the genetic diversity of birds [39,40] for deciphering their evolutionary histories [41,42] and species identification [43,44]. As a genetic marker, *Cytb* has several valuable properties that can be used for inferring different evolutionary and population processes. *Cytb* gene sequence is characterized by both slowly and rapidly evolving codon positions as well as conserved and variable regions, which makes it ideal for deciphering diversity and systematics questions starting from detailed phylogeny [45,46,47,48,49,50] to the population and recent divergence levels [51,52,53,54]. *Cytb* gene variability can be used to identify the signs of adaptive evolution [55] as well as local declines in diversity, which is indicative of selective sweeps [56,57].

The majority of data available for the Griffon vulture and its sister species are based on the variability of *Cytb* and new data about *Cytb* variability in the Serbian Griffon vulture population will further improve our understanding of the population structure and local genetic variability of this significant species. In addition, *Cytb* can be used to decipher if specific adaptations to different climate conditions have emerged in the Griffon vulture populations inhabiting various geographical regions. With the detailed analysis performed in this study, we aim to further determine the status of the Griffon vulture population from Serbia, and its perspective as a source population for further reintroduction efforts in the Balkan Peninsula.

## 2. Materials and Methods

### 2.1. Population Location and Sampling

A representative sample of the Griffon vulture population from Serbia consisted of 58 unrelated birds originating from 58 different nests. Sampling was performed in a period from 2013 to 2020 in the gorge of the Uvac River, which is a part of the “Special nature reserve Uvac” as described in [2]. None of the birds was sacrificed or injured during the sampling process. All the procedures applied in this study were reviewed and approved by the Ministry of Nature Protection of the Republic of Serbia, Ministry of Agriculture, Forestry and Water Management of the Republic of Serbia (323-07-09135/2020-05/1) and the ethical committee of the Institute for Biological Research “Siniša Stanković”, Belgrade (323-09135-2020-05). In addition, the feathers from 12 birds were sampled from the collection of the Natural History Museum of Belgrade (Table 1).

For interpopulation analyses, we collected all available and published *Cytb* data from GenBank that were found in the different Griffon vulture populations (Table S1).

For phylogenetic analyses, we collected all available *Cytb* sequences from the following *Gyps* species, including a number of complete mitogenomes: *G. africanus*, *G. bengalensis*, *G. coprotheres*, *G. f. fulvus*, *G. f. fulvescens*, *G. himalayensis*, *G. indicus*, *G. rueppellii* and *G. tenuirostris* (Table S1).

### 2.2. DNA Extraction and Molecular Genetics Analyses

#### 2.2.1. DNA Extraction from the Contemporary Samples

The DNA extraction was performed using the GeneJET Genomic DNA Purification Kit (Thermo Fisher Scientific Cat.No. K0721) and a modified salting-out protocol. For the salting-out protocol, the blood samples of 20 μL were adjusted to 100 μL using TE buffer and mixed with 300 μL of lysis buffer (10 mM TrisHCl pH 8, 0.4 M NaCl, 2 mM EDTA pH 8) with the addition of 10 μL 20% SDS, 10 μL of Proteinase K (20 mg/mL) and 20 μL of ddH_2_O. The samples were then incubated at 56 °C for two hours in the water bath. After incubation, the 400 μL of 5 M NaCl was added to the mixture, thoroughly vortexed and incubated for 10 min at 4 °C. The DNA was then precipitated with ethanol and dissolved in 50 μL of TE buffer. The quality of the DNA extracts was checked both by the spectrophotometer (NanoPhotometer, IMPLEN, Munich, Germany) and agarose gel electrophoresis.

#### 2.2.2. DNA Extraction from the Museum Samples

To avoid possible contamination, sample preparation and DNA extraction of the museum feather samples was performed in the forensic DNA laboratory in the Center for Forensic and Applied Molecular Genetics at the Faculty of Biology.

DNA extraction was conducted from whole feathers or only the calamus depending on the feather size. Calamus was removed from the feather using a sterile scalpel blade. All feather samples were first rinsed with 3% sodium hypochlorite to remove possible surface contamination and then rinsed twice with DNase/RNase-free distilled water (Nuclease-Free Water, Qiagen, Valencia, CA, USA).

DNA was extracted using the previously described protocol [58] in the final elution volume of 50 µL. Because keratin is the major component of a feather, the lysis buffer was modified by adding dithiothreitol (DTT) [59] with overnight incubation at 37 °C.

### 2.3. Cytb Analysis

*Cytb* was amplified using the GF-L13740/GF-H15014 set of primers (Table 2). Program for the amplification of the *Cytb* consisted of one cycle of initial denaturation at 94 °C for 5 min, after which there were 35 cycles of 35 s at 94 °C, 35 s at the annealing temperature at 55 °C and 90 s at 72 °C. The step of final elongation was performed at 72 °C for 10 min.

Amplification of the target mtDNA region of *Cytb* gene for each museum sample was performed with primer pairs that amplified four overlapping fragments (GF-L13780/H14316, GF-L14290/H14566, GF-L14537/H14819 and GF-L14790/H15014, Table 2) and AmpliTaq Gold Master Mix (Applied Biosystems, Foster City, CA, USA) in a reaction volume of 25 µL. The PCR was conducted in a ProFlex PCR system (Applied Biosystems, Foster City, CA, USA) with the initial denaturation step at 95 °C for 10 min, followed by 35 cycles at 95 °C for 15 s, 55 °C for 30 s and 72 °C for 1 min. The Sanger sequencing of the amplified products was carried out using BigDye Terminator v. 3.1 sequencing kit (Applied Biosystems, Foster City, CA, USA) on the ABI 3130 Genetic Analyzer (Applied Biosystems, Foster City, CA, USA).

Sequencing of contemporary samples was performed by Macrogen Europe using the same set of primers as for the PCR amplification, while the sequencing of museum samples was performed in the Center for Forensic and Applied Molecular Genetics, Faculty of Biology, the University of Belgrade using the same set of primers as for the PCR amplification.

The acquired *Cytb* sequences were aligned with the reference mitogenome NC_036050 [28], and the haplotypes were determined as the differences to the reference mitogenome. All sequences were deposited in GenBank under the following accession numbers: OL962630–OL962691.

### 2.4. Population Genetics

All the *Cytb* sequences used in analyses were truncated to match the length of the shortest available sequence and aligned using MEGA 10.0.4. [60]. The genetic diversity of analyzed populations was estimated using the standard parameters of genetic diversity: number of haplotypes, number of polymorphic sites, haplotype diversity, nucleotide diversity, random match probability (RMP) and mean number of pairwise differences (MPD). The RMP parameter is calculated as the sum of square frequencies, and it expresses the probability that two randomly picked individuals from a population have a matching genotype [61]. MPD represents the measure of differences between all pairs of haplotypes in the sample. Calculations were performed using Arlequin ver. 3.5.2.2 software [62]. The same software was used for assessing genetic differentiation among populations through the analysis of molecular variances (AMOVA) and estimating pairwise population and overall *F_ST_* values. The statistical significance of all performed tests was assessed with 10,000 permutations. The matrix of pairwise population *F_ST_* values was visualized by two-dimensional scaling (non-metric MDS) using PAST 4.03 [63].

### 2.5. Selection Analysis

Nucleotide sequences for all available *Cytb* haplotypes were translated into protein sequences using MEGA 10.0.4, which allowed for the identification of synonymous and nonsynonymous substitutions. In order to evaluate whether these substitutions were under selective pressure, we ran different codon models (SLAC—Single Likelihood Ancestral Counting; FEL—Fixed Effects Likelihood; aBSREL—Adaptive Branch Site Random Effects Likelihood; MEME—Mixed Effects Model of Evolution) using the Datamonkey web designed tool (http://www.datamonkey.org/; accessed on 26 May 2021).

### 2.6. Phylogeography and Phylogeny

Phylogeographic analysis was performed using 128 mtDNA haplotypes found in 13 different Griffon vulture populations (Table S1). The phylogeographic network was constructed using the median-joining method [64] available in the software Network 10.2.0.0 (Fluxus Technology Ltd, Colchester, England), and star contraction for pre-processing [65] and maximum parsimony calculations [66] for post-processing were implemented and all substitutions were weighted equally since no data of different evolutionary rate for the nucleotide positions in *Cytb* gene in birds are available. Phylogenetic relationships among detected haplotypes within the Griffon vulture species were tested by the means of Bayesian phylogenetic inference in the BEAST v 1.10.4. [67]. The phylogeny of the *Gyps* genus was tested in order to acquire more precise time divergence estimates of the detected haplotypes; the sequences used for the analysis are listed in Table S1. We used the HKY+G model of sequence evolution, which had the best fit to our dataset as inferred from the Akaike Information Criterion (AIC) value calculated in MEGA 10.0.4. We used the strict molecular clock and three different mutation rates; mutation rates for whole mitogenome *m* = 0.00223 $\times 10^{-6}$ and m = 0.00204 $\times 10^{-6}$ represent the body mass corrected substitution rates using Formula (1) [68]:

(1) $$m=\frac{10^{\left(slope*log10\left(mass\right)+intercept\right)}}{100}$$

In addition to using the estimated mutation rate for *Cytb* in *Accipitriformes*, *m* = 0.00124 $\times 10^{-6}$ [69]. All parameters were sampled once every 1000 steps from 150 million Markov chain Monte Carlo (MCMC) steps. Tracer v.1.7.1 [70] was used to assess acceptable mixing, likelihood stationarity of the MCMC chain and adequate effective sample sizes for each parameter (≥200). The maximum clade credibility tree was created by using 1000 burning states and a posterior probability of 0.98. The phylogenetic tree was then constructed, and its topology was edited using FigTree v1.4.4 software (FigTree, http://tree.bio.ed.ac.uk/software/figtree, accessed on 27 December 2021).

## 3. Results

### 3.1. Sequencing of Contemporary and Museum Samples

Overall, four haplotypes were detected among the analyzed Serbian Griffon samples, including contemporary and museum specimens (Table 3). Sample S02 contains two alleles at positions 14074 and 14075, while sample SD6 has two alleles on position 14560, which is an indication of heteroplasmy. The most frequent haplotype represented in 86.21% of contemporary samples is defined by the substitution at position 14650 (haplotype 14650C), while the second most frequent haplotype, represented in 12.07% of contemporary samples, is defined by the substitutions on positions 14650 and 14820 (Table 3). The haplotype defined by the substitutions on positions 14650 and 14682 is found in only one bird, S033. The DNA extraction from museum samples successfully yielded enough DNA for further analysis in only four samples (Table 1). Two of the successfully analyzed museum samples (Griffon_8 and Griffon_10) were classified into the most common mtDNA haplotype detected in the contemporary Griffon vulture population from Serbia. In the museum sample Griffon_9, two alleles on the position 14859 were detected, which could be the result of a degradation process characteristic for ancient DNA. This damage is known as deamination [71] and is one of the most common damages that cause the transition of cytosine to thymine and uracil [72]. Thus, the mtDNA haplotype detected in sample Griffon_9 is most likely the same haplotype as the one found in Griffon_8 and Griffon_10. The mtDNA haplotype detected in Griffon_2 is defined by substitutions at positions 13915 and 14650, and it is identified as a new haplotype not detected in the contemporary Griffon vulture population from Serbia and other populations used in this analysis.

### 3.2. Parameters of Genetic Diversity

For comparison of the parameters of genetic diversity between different Griffon vulture populations, we used the populations whose samples consisted of at least four birds, which accounted for a total of 115 *Cytb* sequences used in this analysis (Table 4). The obtained results show that the values for different parameters vary in a wide range. The highest values for the parameters of genetic diversity were detected for the Griffon vulture population from Spain, while the population from Serbia exhibited the lowest values for the parameters of genetic diversity. Interestingly, although the Griffon vulture population from South Asia (India and Pakistan) was represented with only four birds, it exhibited the highest value for the haplotype diversity parameter. The RMP value was highest in the population from Cyprus since the all detected haplotypes were identical while the lowest value for parameter RMP was detected for the population from Spain, which is in concordance with the high values detected for other genetic diversity parameters.

### 3.3. Differentiation of Analyzed Populations

**Figure 1:**
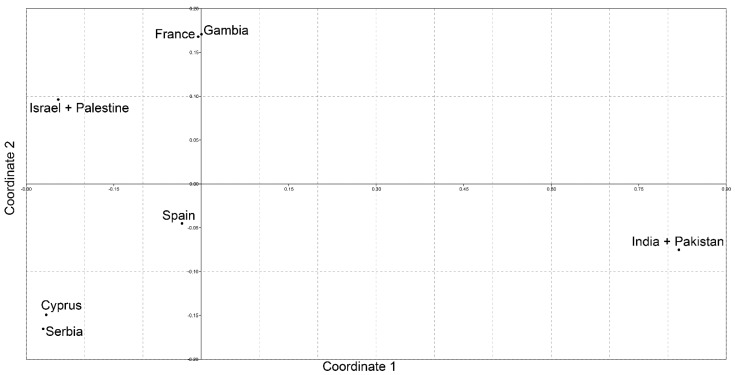
Non-metric multidimensional scaling plot of *F_ST_* distances between the Griffon vulture population of Serbia and other Griffon vulture populations based on the analysis of *Cytb* sequences. The goodness of fit is expressed with the stress value, which is 0.1525 for this data set. Population pairwise *F_ST_* values are presented in Table 6.

AMOVA revealed that 9.61% of the genetic variance can be attributed to the variation among the populations, while the rest of genetic variance can be explained with the variation within populations (Table 5). The AMOVA performed on different groups of populations showed the existence of relatively low but significant value of genetic variance between the groups of populations and the highest detected value, 10.84%, was observed between the groups of populations from southeast Europe with Cyprus (Serbia with Cyprus), southwest Europe with West Africa (Spain and France with Gambia), the Middle East (Israel and Palestine) and South Asia (India and Pakistan) (Table 5). Four tested groups were made according to the geographical proximity of given populations and positioning of the populations on the MDS plot (Figure 1). The AMOVA performed for the four tested groups demonstrated a low percentage of variation between the populations within groups, suggesting that the geographically close populations are genetically more similar to each other than they are to other analyzed populations (Table 5). AMOVA was also performed to test the genetic differentiation of the biggest Griffon vulture populations from the Balkan and the Iberian Peninsulas (Table 5).

The values of pairwise population *F_ST_* between different Griffon vulture populations varied from the highest and statistically significant detected between the pair of populations from Serbia, India and Spain to the lowest and statistically not significant found between the pairs of populations that mostly included Cyprus and Gambia (Table 6). The visualization of pairwise population *F_ST_* matrices with MDS shows the positioning of analyzed populations in two dimensions (Figure 1).

### 3.4. Selection Analysis

Analysis of nucleotide sequence and translation into amino acid sequence showed that the majority of substitutions were silent mutations, while only seven substitutions resulted in the change of primary protein structure (Table 7). The nonsynonymous to synonymous substitution ratio was 0.37, with most substitutions recognized as transitions and one transversion on the position 14879 (Table 7). The selection analysis did not reveal that any of the detected substitutions were under selective pressure and all detected substitutions were categorized as selectively neutral. The same analysis showed that the *Cytb* gene is under strong purifying selection, which eliminates all substitutions that could have a negative effect on the phenotype.

### 3.5. Phylogeography and Phylogeny

**Figure 2:**
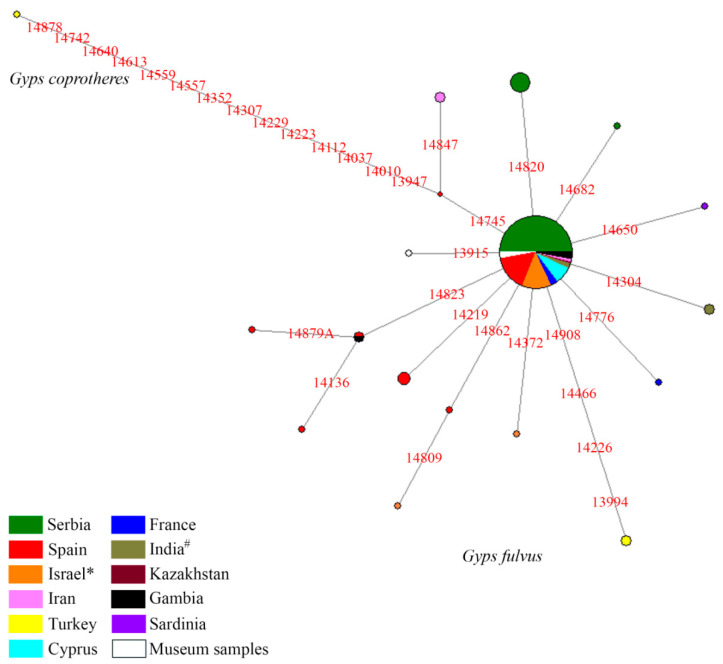
Median-joining phylogeographic network of all mtDNA haplotypes detected in different Griffon vulture populations based on the variability of *Cytb* sequences. The size of the node is proportional to the number of individuals. Differences at nucleotide positions are presented as numbers; transversion is marked with suffix, while the transitions are marked as nucleotide positions. Geographical origin of the samples is shown in the legend; *—including Palestine, ^#^—including Pakistan.

The mtDNA haplotype network representing phylogeographic relationships within Griffon vulture species is presented in Figure 2. The *Cytb* sequence of the Cape vulture (*G. coprotheres*) was used to root the network. A total of 16 different mtDNA haplotypes were detected (excluding the Cape vulture haplotype), defined by 19 polymorphic sites. The network is defined by star-like phylogeny, which indicates rapid and recent diversification. The most frequent mtDNA 14560C haplotype was present in most of the populations included in the analysis, while others were mostly private and represented by one bird in the population. Three different haplotypes were detected in the Griffon vulture population of Serbia, 14560C and two private ones. As expected, the Griffon vulture population from Spain has the highest haplotype diversity of all European populations (Table 4) defined by six haplotypes: haplotype 14560C; four private haplotypes; and the haplotype defined by substitution on position 14823, which is shared with the population of Gambia. The branch defined by the substitution at position 14862 contains two haplotypes, one from Spain and the haplotype from Israel, which has an additional substitution at position 14809. In order to compare the haplotype diversity and distribution in the Griffon vulture with its sister species, we constructed a haplotype network using available *Cytb* sequences for other species from the *Gyps* genus (Figure S1). This analysis showed that the Griffon vulture species has the highest number of *Cytb* haplotypes compared to other *Gyps* species, but also that the pattern of the variability is similar in most of the species of the analyzed genus. The Indian vulture (*G. indicus*), Slender-billed vulture (*G. tenuirostris*) and Himalayan vulture (*G. himalayensis*) exhibit similar patterns of the star-like phylogeny, indicating a recent and rapid diversification, while other *Gyps* species show more stable diversification. A small number of haplotypes and pronounced star-like phylogeny for Indian vulture and Slender-billed vulture is also indicative of recent loss of genetic diversity caused by the significant decrease in the number of birds that were reported for these species. Although the White-rumped vulture (*G. bengalensis*) and White-backed vulture (*G. africanus*) are labelled as “Critically Endangered” by the IUCN Red list because of decreasing numbers, the haplotype network suggests that the mtDNA genetic variability is high and stable.

**Figure 3:**
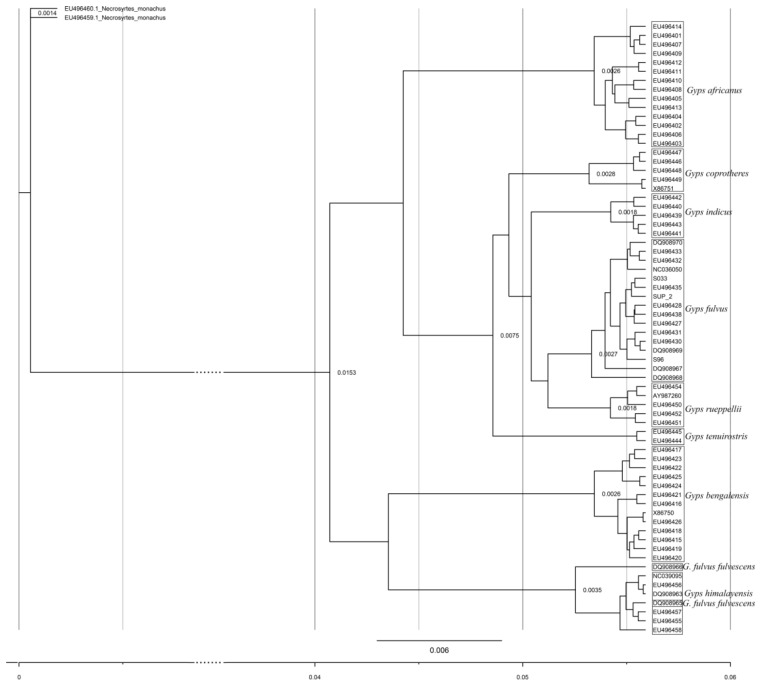
Phylogenetic tree of the *Gyps* genus based on the variability of *Cytb* sequence. Two *Necrosyrthes monachus Cytb* sequences were used as an outgroup in order to root the tree. Heights whose posterior rates were higher than 98% are presented on the nodes. Sequences used for the reconstruction of the phylogenetic tree are presented with their accession numbers and are listed in Table S1.

The *Gyps* genus phylogenetic tree of mtDNA lineages based on Bayesian tree analysis supported by high posterior probability (HPD > 95%) for each node is presented in Figure 3. Each mtDNA lineage is represented with one sequence in order to determine the time divergence of each lineage. Divergence time estimates for whole mitogenome and *Cytb* with 95% HPD interval are presented in Table S2, while the divergence time estimates for each of the mtDNA lineages found in *G. fulvus* species are presented in Figure S2. Phylogeny of mtDNA lineages found in the Griffon vulture population correspond to the haplotype network presented in Figure 1 and the entire phylogeny for *Gyps* genus corresponds to the haplotype network shown in Figure S1. The haplotype defined by substitution on the position 14820 diverged around 358–644 thousand years ago (kya), while the haplotype defined with the substitution at the position 14682 diverged around 179–322 kya (Figure S2). The same haplotype 14682 belongs to the phylogenetic branch together with the haplotype from France and from Griffon_2, which diverged from these two lineages around 269–483 kya (Figure S2). This entire branch diverged around 358–644 kya from the branch that contains the most frequent haplotype found in the *G. fulvus* species (Figure 2 and Figure S1). Based on the phylogenetic analysis, it seems that the divergence of private mtDNA lineages, including the museum sample, found in Serbia could be traced to around 358–644 kya.

## 4. Discussion

Most of the species classified as endangered are facing extinction at a rapid rate today. Species that are of economic importance have a chance to be preserved, and the Griffon vulture is one of those species. Species belonging to the *Gyps* genus are particularly endangered in Asia and Africa, where their extinction poses a threat to the functioning of the grassland communities and migratory ruminants. Evaluating genetic diversity and differentiation of threatened wildlife populations is essential for determining conservation units and developing appropriate conservation and management strategies. Although the Griffon vulture as a species is not itself threatened, some of its populations are under threat of extinction from their natural habitats or have already disappeared. Human-induced declines in numbers and habitat occupancy can lead to the extinction of populations of this species throughout its native range of distribution. Many conservation efforts have been devised for the protection and reintroduction of the endangered Griffon vulture species of the Balkan Peninsula. Based on previous work on genetic variability and population health, the Serbian Griffon vulture population has the potential to be an important source for reintroduction efforts in other continental parts of southeast Europe [2,6]. In this paper, we present the results of the analysis of genetic variability of the most commonly used mtDNA marker in birds, *Cytb*, with the aim to further assess the status of the Serbian Griffon vulture population and its perspectives for conservation efforts in the Balkan Peninsula.

A total of 16 *Cytb* haplotypes defined by 19 polymorphic sites were detected in contemporary Griffon vulture populations and museum samples. The most common 14560C haplotype is present in all analyzed populations with the exception of Turkey and Sardinia, which are represented with only two and one bird, respectively. Analysis of museum samples collected in Serbia showed that the more recent samples, collected between 1980 and 1998, have the most common haplotype prevalent in the contemporary Serbian Griffon population. However, the oldest successfully amplified sample from 1920 has a distinct haplotype never before identified in any analyzed Griffon vulture populations. Loss of genetic diversity and elevated inbreeding levels are expected in populations that experienced serious bottleneck events, such as those that happened with the Serbian Griffon vulture population from 1950 to 1995 [6]. *Cytb* genetic diversity of the contemporary Serbian population with three haplotypes is a strong indication that this population successfully recovered from its previous predicament and is now a strong and stable population. Although it may seem low, the observed haplotype diversity is in concordance with previously published studies regarding mtDNA variability of the entire *Gyps* genus [16,26] and overall higher values of genetic diversity level in *G. fulvus* compared to species with similar biology and ecological niche *Gypaetus barbatus* [73] and *Neophron percnopterus* [74]. The same conclusion can be inferred with our analysis of haplotype diversity of all publicly available *Cytb* sequences within the *Gyps* genus. The greatest number of haplotypes was detected in the *Gyps* vulture, followed by *G. africanus* and *G. bengalensis.* This is not a surprising result bearing in mind that *G. vulture* has the largest area of distribution of all analyzed species [25]. It is interesting to note that the population from South Asia, with only four individuals represented in the sample, has the highest values of genetic diversity represented by haplotype diversity and that the Spanish population, with more than 75% of total Griffon vulture individuals, although high showed lower values for this parameter. However, as expected, overall, the Spanish population harbors the highest genetic diversity among all analyzed populations.

Closer analysis of haplotype distribution within specific populations, with one prevalent and few private haplotypes, is a strong indication of discrete genetic segregation between populations and is further corroborated by analysis of molecular variances. A significant percentage of genetic variance can be attributed to the variation among populations and the position of populations on the MDS plot shows clear differentiation among them as four distinct clusters (southeast Europe with Cyprus, southwest Europe with West Africa, the Middle East and South Asia) can be observed. In addition, when populations with the highest numbers of sequenced individuals, i.e., Serbian and Spanish, were compared, a significantly high 10% of the genetic variance could be explained by the variation among populations. The observed high and statistically significant *F_ST_* value further confirms a pronounced genetic differentiation between these two populations.

The presented results are in concordance with previously reported genetic differentiation based on microsatellite variability between these two populations [2]. It is interesting to note that a similar pattern of genetic differentiation between Iberian and Balkan populations was observed in Eurasian black vulture, where *Cytb* analysis revealed the existence of two distinct evolutionary lineages corresponding to breeding populations in Spain and the Balkans [75]. Considering the fact that, in general, raptor birds have a low level of genetic diversity [76,77,78,79,80,81,82] and that in species with high dispersal capabilities, low values of genetic structuring is expected [83,84], such clear differentiation between the two largest remaining European Griffon vulture populations may seem surprising. Genetic distinctiveness between these populations may be contributed to several factors and probably arise foremost due to the social monogamy and pronounced philopatric behavior of this species and the specific environmental setting characteristic for these two geographic regions that are reflected in slightly different physiology and reproductive phenology. Griffon vulture individuals show high nest fidelity and strong adult natal philopatry, which is presumed to give an advantage in the intra-sexual competition for territories [85,86]. This behavior favors colony formation and reduction in dispersion rate and may have several consequences for the levels of genetic variability in any given population. Strong adult natal philopatry may increase the inbreeding level and lead to the reduction of population genetic variability but, on the other hand, it is considered as a factor that stimulates a rise in frequencies of genetic variants that are better adapted to the specific habitat requirements and lead to the population-specific genetic variability, such as that previously detected in different geographical regions [2,27,28].

The establishment of population-specific genetic variants may be further propelled by social monogamous behavior and multigenerational natal philopatry leading to genetic differentiation between populations, as already shown for the island populations of Crete and Sardinia [27,28]. Although our selection analysis did not reveal the presence of variants favored by selection, and all mutations were defined as neutral, the same analysis showed that the *Cytb* gene is under strong purifying selection, which eliminates all substitutions that could have a negative effect on phenotype, as shown in the case of low-altitude deer mice [87] and four different *Gerbillus* species [55]. The second reason behind genetic differentiation between populations may be specific morphological and reproductive differences in different climates. For example, the Serbian Griffon vulture population is the only continental population in Europe and individuals, on average, have greater body mass and later hatching time than individuals from Spain. Adaptation to harsher continental climate is the most likely explanation of larger body size [88,89] and delay in hatching time is in correlation with the delayed pasturing season in continental compared to Mediterranean climate [3,90,91]. In addition, Griffon vultures marked in the nests in Serbia have been recorded in large numbers during migrations in the Middle East. The absence of migration of young birds from Serbia across the Alps confirms the isolation of the Western (Iberian) from Eastern (Balkan) European populations of the Griffon vultures [92].

The *Gyps* genus phylogenetic tree of *Cytb* lineages based on Bayesian tree analysis is supported by high posterior probability for each node and in concordance with previously published results based on different mtDNA genes [16,25]. Both the haplotype network and phylogeny confirm genealogical relationships between different mtDNA lineages of the same species and between sister species of *Gyps* genus. Haplotype networks also reflect the demographic history of the species indicating a rapid diversification [16], and it can be observed that the species that underwent pronounced demographic decline exhibit lower numbers of haplotypes, as can be seen for Indian vulture and Slender-billed vulture [25]. The origin of the Griffon vulture species is dated to about 2–3 million years ago (Mya) and proposed times of differentiation between private mtDNA lineages obtained from our analysis could be traced to around 358–644 kya.

Population genetic variability of any given species is shaped by its distant and recent history, demography and biogeography. In highly philopatric, gregarious, monogamous species, such as the Griffon vulture, behavior may be a factor that also shapes genetic variability. As already demonstrated in almost all analyzed vulture species, human-induced demographic declines significantly influence standing genetic variation reflected in the loss of diversity, inbreeding depression and presence of population-specific genetic variants [93]. All these must be taken into account when defining the appropriate scale and subunits for conservation management as well as making restocking and population restoration strategies. Large-scale genetic analyses of different markers as well as in situ evaluation of overall general population health must be taken into consideration when conservation strategies are made.

European Griffon vulture populations are the subject of several active restocking programs with variable success (reviewed in [3]). Successful reintroduction in its historic habitats in France encouraged many other conservation and reintroduction initiatives worldwide. The French Griffon vulture population is stable and thriving, and genetically similar to the Spanish population from which most individuals used for restocking descend [5]. However, an example from Sardinia, where the successful reintroduction of individuals of Spanish descent was performed, showed significant change in native genetic diversity due to the import of foreign haplotypes and elicits extreme caution in the selection of source population and individual birds for restocking purposes [3,28]. Finally, failed attempts of repopulation in Bulgaria with individuals from Spain demonstrate that adaptation to territory and environmental conditions such as climate is an important factor that must be taken into consideration when reintroduction efforts are made [3].

## 5. Conclusions

Our analysis of *Cytb* variability in the Serbian Griffon vulture population, along with previously published data [2], is a strong indication that this population is the main source population to be considered for restocking purposes in the Balkan Peninsula.

## Figures and Tables

**Table 1:** Museum samples used in the study with the successful DNA extraction and PCR amplification.

Sample ID	Species	Age of Sample (Year)	Geographical Origin of Sample	Type of Sample	Successful Isolation	Successful PCR
1	*G. fulvus*	1904.	Niš	Feather		
2	*G. fulvus*	1920.	Kupinovo	Feather	+	+
3	*G. fulvus*	1907.	Kučevište	Feather		
4	*G. fulvus*	1938.	Serbia	Feather		
5	*G. fulvus*	1860.	Kopaonik	Feather		
6	*G. fulvus*	1980–1986.	Kragujevac	Feather		
7	*G. fulvus*	1998.	Ložište Čajak	Feather		
8	*G. fulvus*	1998.	Trešnjica	Feather	+	+
9	*G. fulvus*	1980–1990	Serbia	Feather	+	+
10	*G. fulvus*	1980–1990	Serbia	Feather	+	+
11	*G. fulvus*	1980–1990	Serbia	Feather		
12	*G. fulvus*	1980–1990	Serbia	Feather		

**Table 2:** List of primers used for the PCR amplification and sequencing of *Cytb* with the product sizes and annealing temperatures.

Primer Pairs	Product Size (bp)	Tm (°C)
GF-L13740 5′ TAATCAACAACTCCCTAATCGACCTAC 3′ GF-H15014 5′ CCTTTTGGGCCGAGAACTCT 3′	1320	55
GF-L13780 5′ CATTTGATGAAACTTCGGGTC 3′ GF-H14316 5′ GTGAGGGTGGGGTTATCTACG 3′	577	55
GF-L14290 5′ CCATACATCGGACAAACCCTTG 3′ GF-H14566 5′ GCTGGGGTGAAGTTTTCTGG 3′	317	55
GF-L14537 5′ CTCCCATTAACAGCCCTAGC 3′ GF-H14819 5′ CTACTGGCTGGCTGCCGATTC 3′	322	55
GF-L14790 5′ CCCAACTCCTATACTGAACC 3′ GF-H15014 5′ CCTTTTGGGCCGAGAACTCT 3′	263	55

**Table 3:** The mtDNA haplotypes determined by the variability of *Cytb* sequence detected in the Griffon vulture population from Serbia as defined in comparison to reference mitogenome sequence NC_036050. According to IUPAC nomenclature Y stands for C or T on a given nucleotide position.

Sample ID	Haplotype	Positions
Griffon_2 (Museum)	13915T 14650C	13908-14949
Griffon_8, Griffon_10 (Museum)	14650C	13921-14949
Griffon_9 (Museum)	14650C 14859Y	13908-14949
BE423, S00, S01, S02, S03, S25, S26, S27, S28, S31, S32, S33, S34, S43, S46, S51, S53, S54, S069, S69, S70, S72, S74, S078, S87, S88, S89, S94, S95, S99, SA3, SA4, SA6, SA9, SC2, SC3, SC6, SC8, SD4, SD6, SD8, SE0, SE2, SF1, SF4, SF5, SF6, SF7, SF8, SF9	14650C	13908-14949
S09, S96, SC9, SD0, SD1, SE1, SF0	14650C 14820G	13908-14949
S033	14650C 14682G	13908-14949

**Table 4:** Standard parameters of genetic diversity based on the variability of *Cytb* sequence.

Population	N	H	Nps	Hd	π	RMP	MPD
Serbia	58	3	2	0.2462 ± 0.0686	0.000245 ± 0.000310	0.7580	0.25 ± 0.29
Israel *	14	3	3	0.2747 ± 0.1484	0.000419 ± 0.000450	0.7449	0.43 ± 0.41
India ^#^	4	2	1	0.6667 ± 0.2041	0.000658 ± 0.000739	0.5000	0.67 ± 0.63
France	4	2	1	0.5000 ± 0.2652	0.000488 ± 0.000605	0.6250	0.50 ± 0.52
Spain	23	6	5	0.5138 ± 0.1199	0.000718 ± 0.000617	0.5085	0.73 ± 0.57
Cyprus	8	1	0	0.0000 ± 0.0000	0.000000 ± 0.000000	1.0000	0.00 ± 0.00
Gambia	4	2	1	0.5000 ± 0.2652	0.000494 ± 0.000612	0.6250	0.50 ± 0.52

N—number of individuals, H—number of haplotypes, Nps—number of polymorphic sites, Hd—haplotype diversity, π—nucleotide diversity, RMP—random match probability, MPD—mean number of pairwise differences, *—including Palestine, ^#^—including Pakistan.

**Table 5:** Outcomes of AMOVA analysis based on the variability *Cytb* gene analyzed in: (**a**) 7 populations, (**b**) four groups of populations and (**c**) two populations.

**(a)**				
**Source of Variation**	**Df**	**Sum of Squares**	**Variance Components**	**Percentage of Variation**
Among Populations	6	2.724	0.02020	**9.61 (*p* = 0.00436)**
Within Populations	108	20.511	0.18991	90.39
Total	114	23.235	0.21011	
**(b)**				
**Four Groups of Populations Source of Variation**	**df**	**Sum of Squares**	**Variance Components**	**Percentage of Variation**
Among Groups	3	2.142	0.02314 Va	**10.84 (*p* = 0.02703)**
Among Populations Within Groups	3	0.582	0.00046 Vb	0.21 (*p* = 0.65584)
Within Populations	108	20.511	0.18991 Vc	**88.95 (*p* = 0.00356)**
Total	114	23.235	0.21351	
**(c)**				
**Source of Variation (Serbia vs. Spain)**	**df**	**Sum of Squares**	**Variance Components**	**Percentage of Variation**
Among Populations	1	0.899	0.02143	**10.01 (*p* = 0.00228)**
Within Populations	79	15.225	0.19272	89.99
Total	80	16.123	0.21415	

d.f.—degrees of freedom, *p*—statistical significance (statistically significant values are bold).

**Table 6:** Pairwise population *F_ST_* (below diagonal) and *F_ST_ p* values (above diagonal and italic) between the populations based on the sequence variability in *Cytb* gene found in seven different *G. fulvus* populations. Significant *F_ST_* values (*p* ≤ 0.05) are in bold letters.

	Serbia	Israel *	India ^#^	France	Spain	Cyprus	Gambia
Serbia	-	*0.05782*	***0.00673***	*0.21740*	***0.00188***	*0.64489*	*0.17117*
Israel *	0.05844	-	***0.04109***	*0.55064*	*0.16691*	*0.99990*	*0.56430*
India ^#^	**0.45734**	**0.28568**	-	*0.43857*	*0.07791*	*0.08969*	*0.43590*
France	0.13839	0.01510	0.22222	-	*0.52955*	*0.33640*	*0.99990*
Spain	**0.10007**	0.02064	0.20113	−0.00218	-	*0.46877*	*0.99990*
Cyprus	−0.00451	−0.04523	0.51515	0.18644	−0.01323	-	*0.34155*
Gambia	0.13839	0.01510	0.22222	0.00000	−0.10428	0.18644	-

*—including Palestine, ^#^—including Pakistan.

**Table 7:** Positions of nucleotide substitutions, type of mutations and amino acid change in primary protein structure.

Nucleotide Position	Type of Mutation	Amino Acid Change
T14372C	Transition Nonsynonymous	Val 189 Ala
A14304G	Transition Synonymous	No change
C13994T	Transition Nonsynonymous	Ala 63 Val
C14908T	Transition Nonsynonymous	Pro 368 Ser
C14776T	Transition Nonsynonymous	Leu 324 Ile
C14847T	Transition Synonymous	No change
A14809G	Transition Synonymous	No change
C14862T	Transition Synonymous	No change
C14823T	Transition Synonymous	No change
C14879A	Transversion Nonsynonymous	Thr 358 Asn
T14136C	Transition Synonymous	No change
C14823T	Transition Synonymous	No change
A14219G	Transition Nonsynonymous	Gln 138 Pro
T14650C	Transition Synonymous	No change
A14304G	Transition Synonymous	No change
C14847T	Transition Synonymous	No change
A14820G	Transition Synonymous	No change
A14682G	Transition Synonymous	No change
C13915T	Transition Nonsynonymous	Leu 37 Phe

## Data Availability

Sequences generated in this work were deposited in GenBank under the following accession numbers: OL962630-OL962691.

## Supplementary Materials

The following supporting information can be downloaded at: https://www.mdpi.com/article/10.3390/life12020164/s1, Table S1: List of Griffon vulture *Cytb* sequences; Table S2: Time estimates of the divergence of different mtDNA lineages in Gyps genus; Figure S1: Median-joining phylogeographic network for *Gyps* genus; Figure S2: Simplified phylogenetic tree of Gyps genus.
