# Supplementary material for: Genetic Diversity Analysis of Mitochondrial Cytb Gene, Phylogeny and Phylogeography of Protected Griffon Vulture (Gyps fulvus) from Serbia
Source: Life (Basel). 2022 Jan 22;12(2):164. doi: 10.3390/life12020164 (PMC8880743; doi:10.3390/life12020164)
Supplement: Supplementary file 1 [file life-12-00164-s001.zip › Figures S1 and S2.pdf]

life

Supplementary Materials: Genetic Diversity Analysis of Mitochondrial Cytb Gene, Phylogeny and Phylogeography of Protected Griffon Vulture (Gyps fulvus) from Serbia

Slobodan Davidović [1,*], Saša Marinković [2], Mila Kukobat [3], Milica Mihajlović [4], Vanja Tanasić [4], Irena Hribšek [5], Marija Tanasković [1] and Marina Stamenković-Radak [1,3]

[1] Department of Genetics of Populations and Ecogenotoxicology, Institute for Biological Research "Siniša Stanković"—National Institute of the Republic of Serbia, University of Belgrade, Bulevar Despota Stefana 142, 11060 Belgrade, Serbia; marija.tanaskovic@ibiss.bg.ac.rs (M.T.); marina@bio.bg.ac.rs (M.S.-R.)
[2] Department of Ecology, Institute for Biological Research "Siniša Stanković"—National Institute of Republic of Serbia, University of Belgrade, Bulevar Despota Stefana 142, 11060 Belgrade, Serbia; grifon@ibiss.bg.ac.rs
[3] Faculty of Biology, University of Belgrade, Studentski trg 16, 11000 Belgrade, Serbia; mmkukobat@gmail.com
[4] Center for Forensic and Applied Molecular Genetics, Faculty of Biology, University of Belgrade, Studentski trg 16, 11000 Belgrade, Serbia; milica.mihajlovic@bio.bg.ac.rs (M.M.); vanja.tanasic@bio.bg.ac.rs (V.T.)
[5] Birds of Prey Protection Foundation, Bulevar Despota Stefana 142, 11060 Belgrade, Serbia; irena.hribsek@nhmbeo.rs
* Correspondence: slobodan.davidovic@ibiss.bg.ac.rs

Supplementary Materials

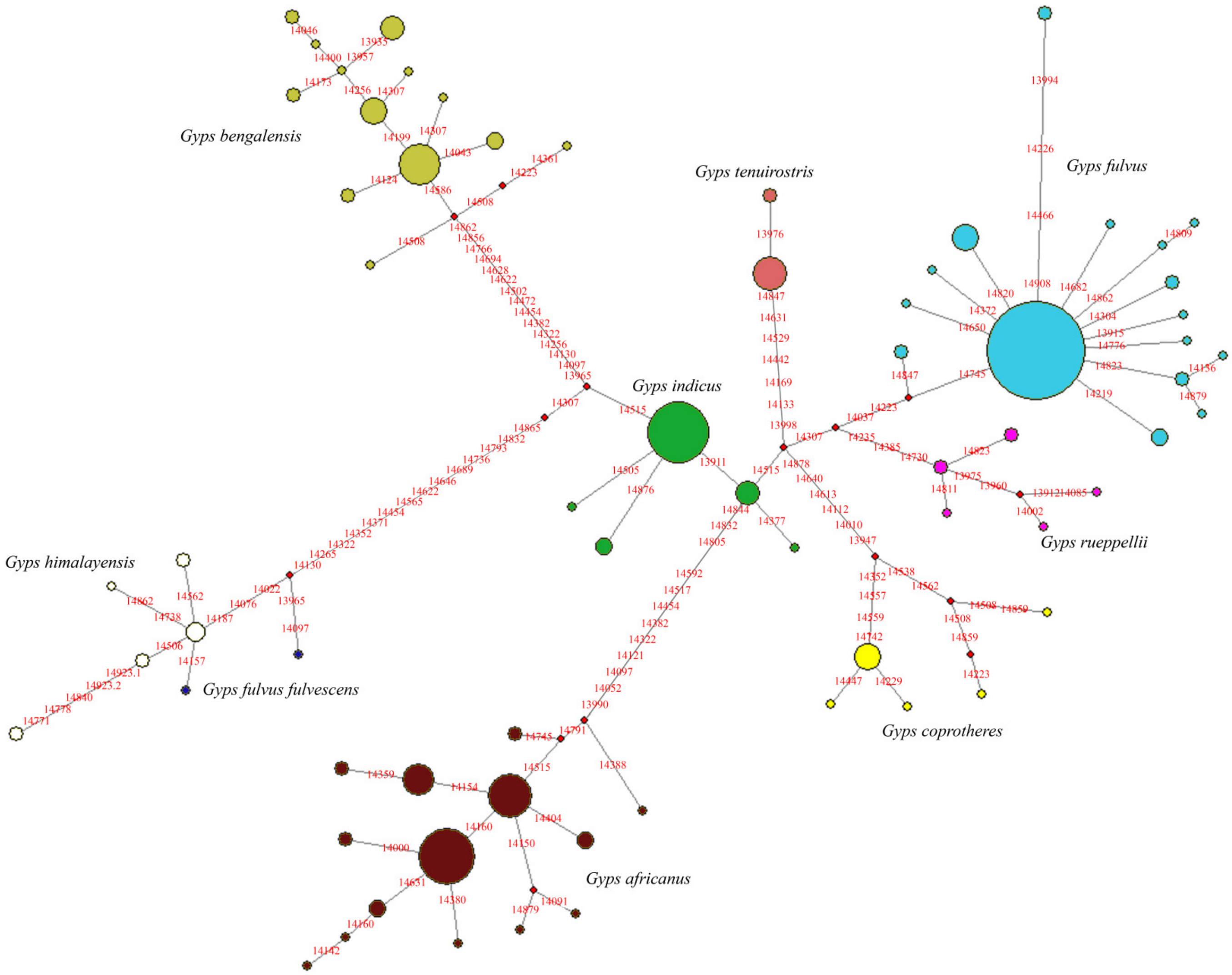

Figure S1. Median-Joining phylogeographic network of all mtDNA haplotypes detected in *Gyps* genus based on the vari-ability of *Cytb* sequences. The size of the node is proportional to the number of individuals. Differences at nucleotide po-sitions are presented as numbers. Different species of *Gyps* genus are presented with unique colours.

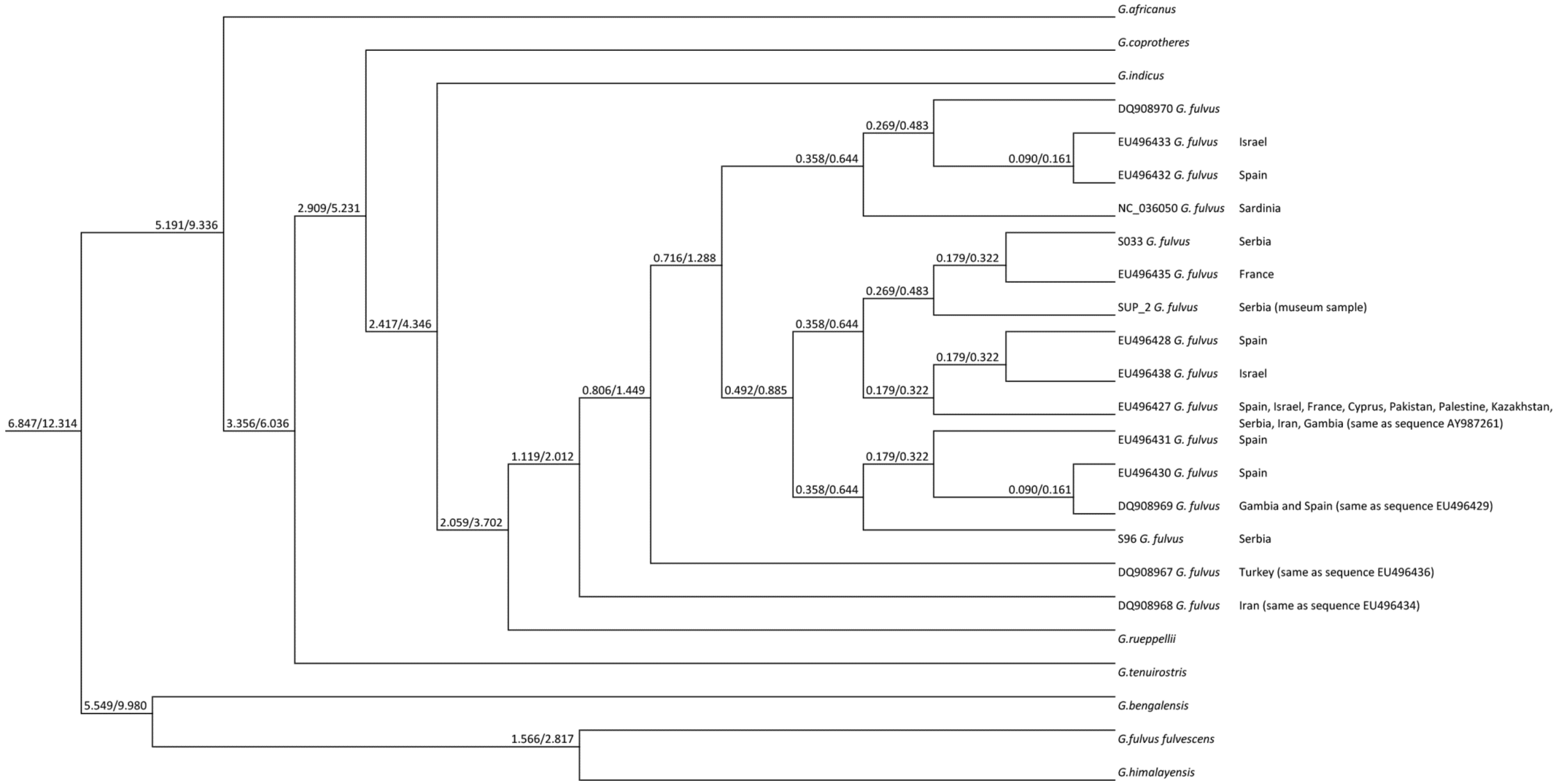

Figure S2. Simplified phylogenetic tree of *Gyps* genus with presented individual mtDNA lineages found in *Gyps fulvus* species. Presented time estimates are on the scale of million years ago (Mya) and are calculated according to node ages using mutation rates for complete mitogenome (0.00223) and *Cytb* gene (0.00124). Each mtDNA linaege found in *Gyps fulvus* spe-cies is presented with one sequence in the tree with additional information of the geographical origin of the sample and reported identical seqeunce found in GeneBank.
