# Supplementary material for: Genetic Diversity Analysis of Mitochondrial Cytb Gene, Phylogeny and Phylogeography of Protected Griffon Vulture (Gyps fulvus) from Serbia
Source: Life (Basel). 2022 Jan 22;12(2):164. doi: 10.3390/life12020164 (PMC8880743; doi:10.3390/life12020164)
Supplement: Supplementary file 1 [file life-12-00164-s001.zip › Tables S1 and S2.pdf]

life

Supplementary Materials: Genetic Diversity Analysis of Mitochondrial Cytb Gene, Phylogeny and Phylogeography of Protected Griffon Vulture (Gyps fulvus) from Serbia

Slobodan Davidović [1,*], Saša Marinković [2], Mila Kukobat [3], Milica Mihajlović [4], Vanja Tanasić [4], Irena Hribšek [5], Marija Tanasković [1] and Marina Stamenković-Radak [1,3]

1 Department of Genetics of Populations and Ecogenotoxicology, Institute for Biological Research "Siniša Stanković"—National Institute of the Republic of Serbia, University of Belgrade, Bulevar Despota Stefana 142, 11060 Belgrade, Serbia; marija.tanaskovic@ibiss.bg.ac.rs (M.T.); marina@bio.bg.ac.rs (M.S.-R.)
2 Department of Ecology, Institute for Biological Research "Siniša Stanković"—National Institute of Republic of Serbia, University of Belgrade, Bulevar Despota Stefana 142, 11060 Belgrade, Serbia; grifon@ibiss.bg.ac.rs
3 Faculty of Biology, University of Belgrade, Studentski trg 16, 11000 Belgrade, Serbia; mmkukobat@gmail.com
4 Center for Forensic and Applied Molecular Genetics, Faculty of Biology, University of Belgrade, Studentski trg 16, 11000 Belgrade, Serbia; milica.mihajlovic@bio.bg.ac.rs (M.M.); vanja.tanasic@bio.bg.ac.rs (V.T.)
5 Birds of Prey Protection Foundation, Bulevar Despota Stefana 142, 11060 Belgrade, Serbia; irena.hribsek@nhmbeo.rs
* Correspondence: slobodan.davidovic@ibiss.bg.ac.rs

Table S1. List of Griffon vulture Cytb sequences used for the phylogeographic analysis and other Gyps species used for the phylogenetic analyses.

Accession number	Species	Region	Population	N	Reference
EU496436	*Gyps f. fulvus*	Middle East	Turkey	1	[1]
DQ908967	*Gyps f. fulvus*	Middle East	Turkey	1	[2]
EU496427	*Gyps f. fulvus*	Middle East	Cyprus	8	[1]
EU496427	*Gyps f. fulvus*	Middle East	Israel	11	[1]
EU496438	*Gyps f. fulvus*	Middle East	Israel	1	[1]
EU496433	*Gyps f. fulvus*	Middle East	Israel	1	[1]
AY987261	*Gyps f. fulvus*	Middle East	Palestine/Israel	1	[2]
X86752	*Gyps f. fulvus*	Middle East	Saudi Arabia	1	[3]
EU496434	*Gyps f. fulvus*	Middle East	Iran	1	[1]
AY987261	*Gyps f. fulvus*	Middle East	Iran	1	[2]
DQ908968	*Gyps f. fulvus*	Middle East	Iran	1	[2]
EU496427	*Gyps f. fulvus*	Western Europe	France*	3	[1]
EU496435	*Gyps f. fulvus*	Western Europe	France*	1	[1]
EU496427	*Gyps f. fulvus*	Southwestern Europe	Spain	15	[1]
EU496428	*Gyps f. fulvus*	Southwestern Europe	Spain	3	[1]
EU496432	*Gyps f. fulvus*	Southwestern Europe	Spain	1	[1]
EU496431	*Gyps f. fulvus*	Southwestern Europe	Spain	1	[1]
EU496430	*Gyps f. fulvus*	Southwestern Europe	Spain	1	[1]
EU496429	*Gyps f. fulvus*	Southwestern Europe	Spain	1	[1]
AY987261	*Gyps fulvus*	Southwestern Europe	Spain	1	[2]
NC036050	*Gyps f. fulvus*	Southwestern Europe	Sardinia	1	[4]
AY987261	*Gyps f. fulvus*	South Asia	Pakistan	2	[2]
DQ908970	*Gyps f. fulvus*	South Asia	India	1	[2]
EU496437	*Gyps f. fulvus*	South Asia	India	1	[1]
AY987261	*Gyps f. fulvus*	Central Asia	Kazakhstan	1	[2]
AY987261	*Gyps f. fulvus*	Africa	Gambia	3	[2]
DQ908969	*Gyps f. fulvus*	Africa	Gambia	1	[2]
DQ908966	*Gyps f. fulvescens*	South Asia	India	1	[2]
DQ908965	*Gyps f. fulvescens*	South Asia	Nepal	1	[2]

DQ908972	*Gyps africanus*	Africa	Africa	1	[2]
AY987263	*Gyps africanus*	Africa	Africa	1	[5]
EU496401	*Gyps africanus*	Africa	Namibia	32	[1]
EU496402	*Gyps africanus*	Africa	Namibia	19	[1]
EU496403	*Gyps africanus*	Africa	Namibia	10	[1]
EU496404	*Gyps africanus*	Africa	Namibia	3	[1]
EU496407	*Gyps africanus*	Africa	Namibia	2	[1]
EU496405	*Gyps africanus*	Africa	Namibia	2	[1]
EU496406	*Gyps africanus*	Africa	Namibia	2	[1]
EU496413	*Gyps africanus*	Africa	Namibia	1	[1]
EU496412	*Gyps africanus*	Africa	Namibia	1	[1]
EU496411	*Gyps africanus*	Africa	Namibia	1	[1]
EU496410	*Gyps africanus*	Africa	Namibia	1	[1]
EU496409	*Gyps africanus*	Africa	Namibia	1	[1]
EU496408	*Gyps africanus*	Africa	Namibia	1	[1]
EU233137	*Gyps africanus*	Africa	Namibia	1	Wink 2007
EU496414	*Gyps africanus*	Africa	South Africa	1	[1]
X86748	*Gyps africanus*	Africa	South Africa	1	[3]
EU496426	*Gyps bengalensis*	South Asia	India	1	[1]
X86750	*Gyps bengalensis*	South Asia	India	1	[3]
EU496415	*Gyps bengalensis*	South Asia	Pakistan	14	[1]
EU496416	*Gyps bengalensis*	South Asia	Pakistan	5	[1]
DQ908977	*Gyps bengalensis*	South Asia	Pakistan	2	[2]
EU496424	*Gyps bengalensis*	South Asia	Pakistan	1	[1]
EU496423	*Gyps bengalensis*	South Asia	Pakistan	1	[1]
EU496421	*Gyps bengalensis*	South Asia	Pakistan	1	[1]
EU496420	*Gyps bengalensis*	South Asia	Pakistan	1	[1]
EU496419	*Gyps bengalensis*	South Asia	Pakistan	1	[1]
DQ908975	*Gyps bengalensis*	South Asia	Pakistan	1	[2]
AY987259	*Gyps bengalensis*	South Asia	Pakistan	1	[5]
EU496416	*Gyps bengalensis*	South Asia	Nepal	2	[1]
DQ908975	*Gyps bengalensis*	South Asia	Nepal	1	[2]
EU496417	*Gyps bengalensis*	Southeast Asia	Cambodia	3	[1]
DQ908979	*Gyps bengalensis*	Southeast Asia	Cambodia	3	[2]
EU496418	*Gyps bengalensis*	Southeast Asia	Cambodia	2	[1]
EU496415	*Gyps bengalensis*	Southeast Asia	Cambodia	2	[1]
EU496422	*Gyps bengalensis*	Southeast Asia	Cambodia	1	[1]
DQ908978	*Gyps bengalensis*	Southeast Asia	Cambodia	1	[2]
DQ908977	*Gyps bengalensis*	Southeast Asia	Cambodia	1	[2]
DQ908974	*Gyps bengalensis*	Southeast Asia	Cambodia	1	[2]
EU496415	*Gyps bengalensis*	Southeast Asia	Thailand	2	[1]
DQ908977	*Gyps bengalensis*	Southeast Asia	Thailand	1	[2]
EU496425	*Gyps bengalensis*	Southeast Asia	Vietnam	1	[1]
DQ908976	*Gyps bengalensis*	Southeast Asia	Vietnam	1	[2]
EU496449	*Gyps coprotheres*	Africa	South Africa	1	[1]
EU496448	*Gyps coprotheres*	Africa	South Africa	1	[1]
EU496447	*Gyps coprotheres*	Africa	South Africa	1	[1]
EU496446	*Gyps coprotheres*	Africa	South Africa	4	[1]
AY987262	*Gyps coprotheres*	Africa	South Africa	3	[2]
MF683387	*Gyps coprotheres*	Africa	South Africa	1	[6]
X86751	*Gyps coprotheres*	Africa	South Africa	1	[3]
EU496440	*Gyps indicus*	South Asia	India	3	[1]
EU496439	*Gyps indicus*	South Asia	Pakistan	40	[1]
EU496441	*Gyps indicus*	South Asia	Pakistan	3	[1]

EU496440	*Gyps indicus*	South Asia	Pakistan	3	[1]
EU496442	*Gyps indicus*	South Asia	Pakistan	1	[1]
EU496443	*Gyps indicus*	South Asia	Pakistan	1	[1]
DQ908971	*Gyps indicus indicus*	South Asia	India	3	[2]
EU496458	*Gyps himalayensis*	Western Europe	France*	1	[1]
EU496455	*Gyps himalayensis*	Western Europe	France*	1	[1]
EU496456	*Gyps himalayensis*	South Asia	India	1	[1]
DQ908963	*Gyps himalayensis*	South Asia	India	1	[2]
DQ908962	*Gyps himalayensis*	South Asia	India	2	[2]
EU496457	*Gyps himalayensis*	East Asia	Kina	1	[1]
DQ908964	*Gyps himalayensis*	East Asia	Kina	1	[2]
DQ908962	*Gyps himalayensis*	East Asia	Kina	1	[2]
NC_039095	*Gyps himalayensis*	East Asia	Kina	1	[7]
DQ908962	*Gyps himalayensis*	South Asia	Nepal	1	[2]
EU496450	*Gyps rueppellii*	Western Europe	France*	2	[1]
EU496452	*Gyps rueppellii*	Western Europe	France*	1	[1]
EU496454	*Gyps rueppellii*	Africa	Gambia	1	[1]
DQ908973	*Gyps rueppellii*	Africa	Gambia	1	[2]
AY987260	*Gyps rueppellii*	Africa	Gambia	1	[2]
EU496451	*Gyps rueppellii*	Africa	South Africa	1	[1]
EU496444	*Gyps tenuirostris*	South Asia	India	1	[1]
EU496445	*Gyps tenuirostris*	South Asia	India	1	[1]
DQ908961	*Gyps tenuirostris*	South Asia	India	1	[2]
DQ908960	*Gyps tenuirostris*	South Asia	India	5	[2]
DQ908960	*Gyps tenuirostris*	South Asia	Nepal	1	[2]
DQ908960	*Gyps tenuirostris*	Southeast Asia	Thailand	1	[2]
DQ908960	*Gyps tenuirostris*	Southeast Asia	Cambodia	5	[2]

* - kept in captivity in zoo.

Table S2. Time estimates of the divergence of different mtDNA lineages in Gyps genus with 95% HPD. Two different time estimates are presented taking into account different mutation rates for the Cytb and complete mitogenome: I - m=0.00223, II - m= 0.00204 and III – m= 0.00124.

	**Complete mitogenome**						***Cytb***		
	**I**			**II**			**III**		
	**Divergence time (Mya)**	**Lower 95%HPD**	**Upper 95%HPD**	**Divergence time (Mya)**	**Lower 95%HPD**	**Upper 95%HPD**	**Divergence time (Mya)**	**Lower 95%HPD**	**Upper 95%HPD**
G. africanus, G. coprotheres, G. indicus, G. fulvus, G. rueppellii, G. tenuirostris and *G. bengalensis, G. himalayensis, G. fulvus fulvescens*	6.847	5.057	8.816	7.489	5.531	9.643	12.314	9.095	15.855
G. africanus lineages EU496414, EU496401, EU496407, EU496409 and EU496412, EU496411, EU496410, EU496408, EU496405, EU496413, EU496404, EU496402, EU496406, EU496403	1.164	0.537	1.835	1.273	0.587	2.007	2.093	0.966	3.300
G. africanus lineages EU496406 and EU496403	0.179	0	0.448	0.196	0	0.489	0.322	0	0.805
G. coprotheres, G. indicus, G. fulvus, G. rueppellii and	3.356	2.282	4.520	3.671	2.496	4.944	6.036	4.105	8.129

G. tenuirostris									
G. coprotheres lineages EU496447, EU496446, EU496448 and EU496449, X86751	1.253	0.537	2.059	1.371	0.587	2.252	2.254	0.966	3.702
G. coprotheres lineages EU496447, EU496446 and EU496448	0.269	0.045	0.627	0.294	0.049	0.685	0.483	0.080	1.127
G. coprotheres lineages EU496449 and X86751	0.134	0	0.358	0.147	0	0.392	0.241	0	0.644
G. indicus lineages EU496442, EU496440 and EU496439, EU496443, EU496441	0.806	0.179	1.566	0.881	0.196	1.713	1.449	0.322	2.817
G. fulvus lineages NC036050, EU496431, EU496435, EU496428, EU496430, EU496427, EU496432, EU496438, DQ908969, DQ908967, DQ908970, S96, S033, SUP_2, EU496433 and DQ908968	1.208	0.627	1.835	1.322	0.685	2.007	2.173	1.127	3.300
G. rueppellii lineages EU496454, AY987260, EU496450 and EU496451, EU496452	0.806	0.269	1.387	0.881	0.294	1.517	1.449	0.483	2.495
G. rueppellii lineages EU496451 and EU496452	0.269	0	0.582	0.294	0	0.636	0.483	0	1.046
G. tenuirostris lineages EU496444 and EU496445	0.224	0	0.582	0.245	0	0.636	0.402	0	1.046
G. himalayensis and *G. fulvus fulvescens*	1.566	0.671	2.551	1.713	0.734	2.790	2.817	1.207	4.588
G. himalayensis lineages NC039095, EU496456, DQ908963, DQ908965*, EU496457, EU496455 and EU496458	0.582	0.224	1.029	0.636	0.245	1.126	1.046	0.402	1.851
G. himalayensis lineages NC039095 and EU496456, DQ908963	0.179	0	0.403	0.196	0	0.441	0.322	0	0.724
G. bengalensis lineages EU496417, EU496423, EU496422, EU496425, EU496424 and EU496418, EU496421, EU496415, EU496420, EU496419, X86750, EU496426, EU496416	1.164	0.492	1.880	1.273	0.538	2.056	2.093	0.885	3.380
G. bengalensis lineages EU496425 and EU496424	0.134	0	0.403	0.147	0	0.441	0.241	0	0.724
G. bengalensis lineages X86750 and EU496426	0.090	0	0.269	0.098	0	0.294	0.161	0	0.483

*-sequence belongs to G. fulvus fulvescens.

References

1. Arshad, M.; Gonzalez, J.; El-Sayed, A.A.; Osborne, T.; Wink, M. Phylogeny and phylogeography of critically endangered Gyps species based on nuclear and mitochondrial markers. Journal of ornithology **2009**, *150*, pp. 419–430–2009 v.2150 no.2002, doi:10.1007/s10336-008-0359-x.
2. Johnson, J.A.; Lerner, H.R.; Rasmussen, P.C.; Mindell, D.P. Systematics within Gyps vultures: a clade at risk. BMC evolutionary biology **2006**, *6*, 1–12, doi:https://10.1186/1471-2148-6-65.
3. Seibold, I.; Helbig, A.J. Evolutionary history of New and Old World vultures inferred from nucleotide sequences of the mitochondrial cytochrome b gene. Philosophical transactions of the Royal Society of London. Series B, Biological sciences **1995**, *350*, 163–178, doi:10.1098/rstb.1995.0150.
4. Mereu, P.; Satta, V.; Frongia, G.N.; Berlinguer, F.; Muzzeddu, M.; Campus, A.; Decandia, L.; Pirastru, M.; Manca, L.; Naitana, S.; et al. The complete mtDNA sequence of the griffon vulture (Gyps fulvus): Phylogenetic analysis and haplotype frequency variations after restocking in the Sardinian population. Biological Conservation **2017**, *214*, 195–205, doi:https://doi.org/10.1016/j.biocon.2017.08.017.
5. Lerner, H.R.; Mindell, D.P. Phylogeny of eagles, Old World vultures, and other Accipitridae based on nuclear and mitochondrial DNA. Molecular phylogenetics and evolution 2005, 37, 327-346, doi:10.1016/j.ympev.2005.04.010.
6. Adawaren, E.O.; Du Plessis, M.; Suleman, E.; Kindler, D.; Oosthuizen, A.O.; Mukandiwa, L.; Naidoo, V. The complete mitochondrial genome of Gyps coprotheres (Aves, Accipitridae, Accipitriformes): phylogenetic analysis of mitogenome among raptors. PeerJ **2020**, *8*, e10034, doi:10.7717/peerj.10034.
7. Jiang, L.; Peng, L.; Tang, M.; You, Z.; Zhang, M.; West, A.; Ruan, Q.; Chen, W.; Merila, J. Complete mitochondrial genome sequence of the Himalayan Griffon, Gyps himalayensis (Accipitriformes: Accipitridae): Sequence, structure, and phylogenetic analyses. Ecology and evolution **2019**, *9*, 8813–8828, doi:10.1002/ece3.5433.
